# Loss of versican and production of hyaluronan in lung epithelial cells are associated with airway inflammation during RSV infection

**DOI:** 10.1074/jbc.RA120.016196

**Published:** 2020-11-21

**Authors:** Gerald G. Kellar, Kaitlyn A. Barrow, Lucille M. Rich, Jason S. Debley, Thomas N. Wight, Steven F. Ziegler, Stephen R. Reeves

**Affiliations:** 1 Department of Defense, United States Army, Washington, USA; 2 Benaroya Research Institute, Seattle, Washington, USA; 3 Department of Immunology, University of Washington, Seattle, Washington, USA; 4 Center for Immunity and Immunotherapies, Seattle Children’s Research Institute, Seattle, Washington, USA; 5 Division of Pulmonary and Sleep Medicine, Department of Pediatrics, University of Washington, Seattle, Washington, USA

**Keywords:** cell adhesion, epithelial cell, extracellular matrix, fibroblast, hyaluronan, myeloid cell, viral immunology, versican (VCAN), ACK, ammonium-chloride-potassium, ALI, air-liquid interface, BALF, bronchoalveolar lavage fluid, BEC, bronchial epithelial cell, CCL, chemokine (C-C motif) ligand, ECM, extracellular matrix, ELISA, enzyme-linked immunosorbent assay, EO, eosinophil, FGM, Fibroblast Growth Media, GAG, glycosaminoglycan, HA, hyaluronan, HAS, hyaluronan synthase, HMW-HA, high molecular weight HA, HLF, human lung fibroblast, HYAL, hyaluronidase, LMW-HA, lower molecular weight HA, MMP, matrix metalloproteinase, MO, monocyte, PMN, polymorphonuclear neutrophils, poly I:C, polyinosinic:polycytidylic acid, RSV, espiratory syncytial virus, SPC-Cre, surfactant protein-C Cre, TLR-3, toll-like receptor 3, TSG-6, TNFα-stimulated gene-6

## Abstract

Airway inflammation is a critical feature of lower respiratory tract infections caused by viruses such as respiratory syncytial virus (RSV). A growing body of literature has demonstrated the importance of extracellular matrix changes such as the accumulation of hyaluronan (HA) and versican in the subepithelial space in promoting airway inflammation; however, whether these factors contribute to airway inflammation during RSV infection remains unknown. To test the hypothesis that RSV infection promotes inflammation *via* altered HA and versican production, we studied an *ex vivo* human bronchial epithelial cell (BEC)/human lung fibroblast (HLF) coculture model. RSV infection of BEC/HLF cocultures led to decreased hyaluronidase expression by HLFs, increased accumulation of HA, and enhanced adhesion of U937 cells as would be expected with increased HA. HLF production of versican was not altered following RSV infection; however, BEC production of versican was significantly downregulated following RSV infection. *In vivo* studies with epithelial-specific versican-deficient mice [SPC-Cre(+) *Vcan*^−/−^] demonstrated that RSV infection led to increased HA accumulation compared with control mice, which also coincided with decreased hyaluronidase expression in the lung. SPC-Cre(+) *Vcan*^−/−^ mice demonstrated enhanced recruitment of monocytes and neutrophils in bronchoalveolar lavage fluid and increased neutrophils in the lung compared with SPC-Cre(−) RSV-infected littermates. Taken together, these data demonstrate that altered extracellular matrix accumulation of HA occurs following RSV infection and may contribute to airway inflammation. In addition, loss of epithelial expression of versican promotes airway inflammation during RSV infection further demonstrating that versican’s role in inflammatory regulation is complex and dependent on the microenvironment.

Respiratory syncytial virus (RSV) is a major causative pathogen leading to lower respiratory tract infections in young children and is associated with substantial morbidity and increased health care burden (1). Each year in the United States approximately 800,000 infants require medical evaluation and 2% to 3% of all infants require hospitalization in their first year of life as a result of RSV bronchiolitis (2). Throughout the world, RSV is the leading cause of hospital admissions for infants and accounts for approximately 239,000 deaths in children under the age of 5 years annually (3, 4). Although most infants do not develop bronchiolitis severe enough to warrant inpatient treatment, exposure to RSV is highly prevalent, with nearly all children demonstrating evidence of prior RSV infection by 2 years of age (5). Beyond the major public health concerns associated with RSV infections in infancy, RSV infections have also been identified as a significant risk factor for the subsequent development of asthma in school-aged children (6). Prior studies have highlighted that ciliated bronchial epithelial cells (BECs) are the primary target for RSV lower respiratory tract infections; however, the mechanisms driving RSV recruitment of leukocytes are incompletely understood and remain an active area of investigation (7, 8, 9, 10).

To date, many of the existing studies of airway inflammation during infections have focused on the production of cytokines. More recently, there has also been an increasing appreciation for the role of the extracellular matrix (ECM) in leukocyte trafficking and modulation of local airway inflammation (11, 12, 13, 14). One important ECM constituent that has been identified for its unique ability to modulate inflammatory responses is the glycosaminoglycan (GAG) hyaluronan (HA) (15, 16, 17). In both acute and chronic inflammation, HA accumulation in the lung increases and directly affects the regulation of inflammatory processes and tissue repair (14, 16, 17, 18, 19, 20). Evidence from both animal and human cell culture models has demonstrated that increased accumulation of HA occurs during viral infection and/or treatment with viral mimetics (21, 22). In *in vivo* studies, HA levels in lung tissue and bronchoalveolar lavage fluid (BALF) are elevated and correlate with the degree of airway inflammation in murine models of asthma (23, 24). Recently, our group has reported that RSV infection of human lung fibroblasts (HLFs) leads to the formation of an ECM that is enriched with HA, which displays greater adhesion of mast cells and directly enhances the expression of mast cell proteases through contact with the ECM post RSV infection (25).

Beyond the direct contributions of HA in the regulation of inflammation and tissue repair following injury, HA also interacts with other important ECM constituents that serve to regulate the adhesion, retention, migration, and activation of leukocytes, such as versican (reviewed in 26). Versican is a large ECM proteoglycan that exists in multiple isoforms each comprising a core protein and variable lengths of chondroitin sulfate GAG chains that are generated *via* alternative splicing of the versican exons (27). Versican isoforms are expressed broadly throughout human tissue with V0, V1, V2, and V3 isoforms all expressed in the lung (28) and V0, V1, and V3 expressed in BECs (29). In a number of diseases including those of the lung, versican is upregulated and accumulates during inflammation and is thought to play an important role in the modulation of the inflammatory process (26). For example, studies in cultured HLFs treated with the viral mimetic polyinosinic:polycytidylic acid (poly I:C), a toll-like receptor 3 agonist, demonstrated enhanced versican accumulation that led to the formation of an ECM that enhanced monocyte binding (21). Subsequent *in vivo* studies in tamoxifen-inducible versican-deficient mice demonstrated that lungs from versican-deficient mice did not display enhanced accumulation of HA or versican in the perivascular and peribronchial regions following poly I:C administration compared with littermate controls. Furthermore, the versican-deficient mice did not display increased airway inflammatory recruitment and elevated proinflammatory cytokines that were characteristic of poly I:C treatment in the control mice (30). Taken together, these data suggest that, in addition to HA, versican is also likely to play an important role in the recruitment of inflammatory cells during acute respiratory viral illness.

The study of the regulation of HA and versican during acute RSV infection is difficult to accomplish in children given that this would necessitate obtaining biopsy tissue from donor children who are acutely ill with RSV and bronchoscopy is not routinely indicated in the clinical management of children with RSV bronchiolitis. The dynamics between immune cells and the ECM are poorly understood, with the moment-to-moment interactions that take place between the two *in vivo* difficult to truly appreciate. However, our group has extensive experience in both *ex vivo* cell culture models of differentiated, primary human BECs obtained from pediatric donors as well as *in vivo* murine models of lung disease. Both models recapitulate many of the features of human respiratory disease and when used in concert provide a more complete picture of lung pathology.

Based on the findings of prior studies, we hypothesized that RSV infection would lead to greater accumulation of HA and versican, which would in turn enhance the recruitment of leukocytes and promote inflammation during RSV infection. To test the hypothesis that alterations in HA and versican accumulation are important contributors to the inflammatory response during RSV infection, we first employed an *ex vivo* human BEC-HLF coculture model system to examine the effects of RSV infection of BECs on the production of HA and versican by cocultured HLFs. We found that HLFs cocultured with BECs that were infected with RSV displayed increased HA accumulation and enhanced monocyte binding. In contrast, we observed no differences in HLF expression of versican. Interestingly, RSV infection of primary human BECs led to a downregulation of versican expression, which was not anticipated. To further characterize the role of BEC-derived versican, we performed *in vivo* studies to examine the effects of RSV infection on the ECM and inflammatory changes in an airway epithelial-specific versican-deficient mouse model. Our findings demonstrate that HA accumulation is increased and correlates with airway inflammation during RSV infection. Furthermore, epithelial-specific versican-deficient mice demonstrated enhanced airway inflammation, altered HA degradation capability, and increased expression of cytokines and chemokines in response to RSV infection. These findings suggest that the role of versican during RSV infection may be more complex and context dependent.

## Results

### Coculture of HLFs with BECs following RSV infection leads to increased accumulation of HA in the ECM and enhanced leukocyte retention

**Figure 1:**
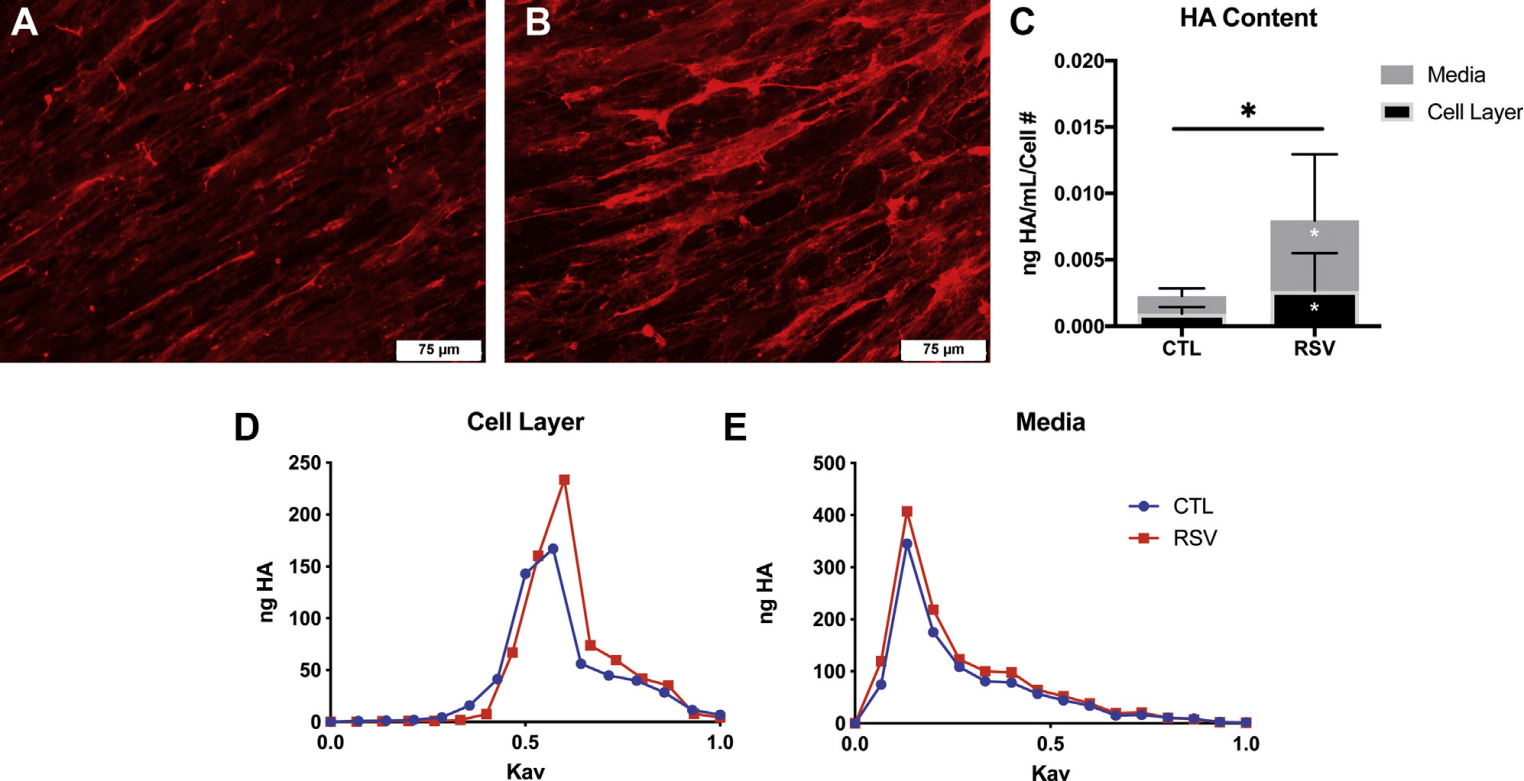
**Analysis of hyaluronan (HA) accumulation by human lung fibroblasts (HLFs) following 96 h of coculture with bronchial epithelial cells (BECs) infected with respiratory syncytial virus** (**RSV)**. *A–B*, immunofluorescence staining for HA in PBS-treated and RSV-infected BECs. HLFs cocultured with RSV-infected BECs displayed greater HA staining and greater HA concentration measured by ELISA in the cell layer and media (*C*). *D–E*, representative chromatograms are depicted illustrating HA size for the cell layer and culture media of HLFs cocultured with BECs with or without RSV infection; no significant differences in the HA hydrodynamic size were evident between treatment groups. (∗*p* < 0.05; n = 7 per group).

To evaluate the effect of RSV infection on the establishment of a HA-enriched ECM in our *ex vivo* BEC/HLF coculture system, HLFs were cocultured with BECs during RSV or mock infection for 96 h. The 96-h timepoint was chosen based on preliminary time course studies (data not shown) and also corresponds with the typical peak of symptoms in children following RSV infection (1). Immunofluorescent staining patterns demonstrated qualitatively greater HA staining in HLFs that were cocultured with RSV-infected BECs compared with coculture with uninfected BECs (Fig. 1, *A–B*). Quantitative analysis of HA content in the HLF cell layer and in the cell culture media also demonstrated greater accumulation of HA in both compartments. Total HA concentration was 3.5-fold greater in HLFs cocultured with RSV-infected BECs compared with controls (Fig. 1*C*; *p* < 0.05) with a 2.8-fold increase in HA concentration in the HLF cell layer (*p* < 0.05) and a 4.0-fold increase in HA concentration in the culture media (*p* < 0.05). In order to compare the distribution of HA hydrodynamic size, equal amounts of HA from cocultured HLF cell layer and media samples were subjected to size exclusion chromatography on an S-1000 column. Media samples from both infected and uninfected BEC-HLF cocultures demonstrated a high-molecular-weight (HMW)-HA profile with virtually no differences between groups. HLF cell layer samples from both HLFs cocultured with control BECs and RSV-infected BECs displayed a more intermediate to lower-molecular-weight (LMW)-HA species distribution compared with media samples. Similar to the media samples, the cell layer HA size profile was not remarkably different between the groups. Representative chromatograms are shown in Figure 1, *D–E*. Taken together, these data demonstrate that HLFs cocultured with RSV-infected BECs produce an ECM enriched with greater quantities of HA without significant differences in the HA size distribution.

**Figure 2:**
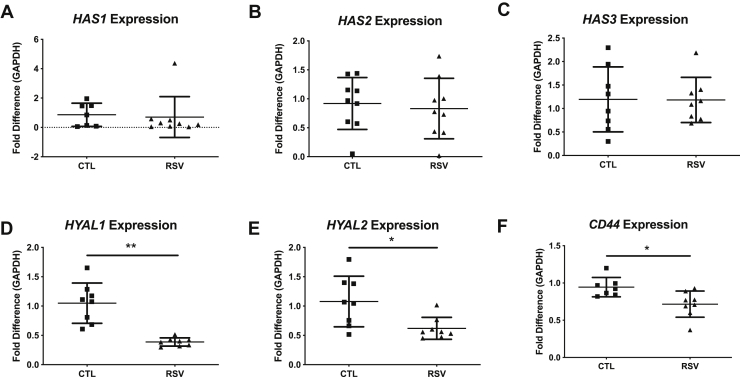
**Gene expression of hyaluronan synthase (*HAS*) and hyaluronidase (*HYAL*) isoforms by human lung fibroblasts from respiratory syncytial virus (RSV)-infected bronchial epithelial cells coculture.** There was no difference in the expression of *HAS1*, HAS*2*, and HAS*3* (*A–C*) in human lung fibroblasts cocultured with RSV-infected or -uninfected bronchial epithelial cells, whereas the expression of *HYAL1*, *HYAL2*, and CD44 (*D–F*) was significantly decreased (∗*p* < 0.05, ∗∗ *p* < 0.01; n = 8 per group).

To evaluate expression patterns of HA-related genes, BEC-HLF cocultures were established and HLFs were harvested following 96 h of coculture. Messenger RNA expression of hyaluronan synthase-1 (*HAS1*), *HAS2*, and *HAS3* was not significantly different by HLFs cocultured with BECs infected with RSV compared with HLFs cocultured with uninfected BECs (Fig. 2, *A*–*C*). However, expression of HA turnover mediators was reduced in HLFs cocultured with BECs infected with RSV compared with controls including decreased expression of hyaluronidase-1 (*HYAL1*) (Fig. 2*D*; 63% reduction, *p* < 0.01), *HYAL2* (Fig. 2*E*; 45% reduction, *p* < 0.05), and *CD44* (Fig. 2*F*; 28% reduction, *p* < 0.05). These data suggest that increased accumulation of HA by HLFs cocultured with BECs infected with RSV is attributable to downregulation of HA turnover mechanisms allowing for the persistence of HA deposited in the ECM.

**Figure 3:**
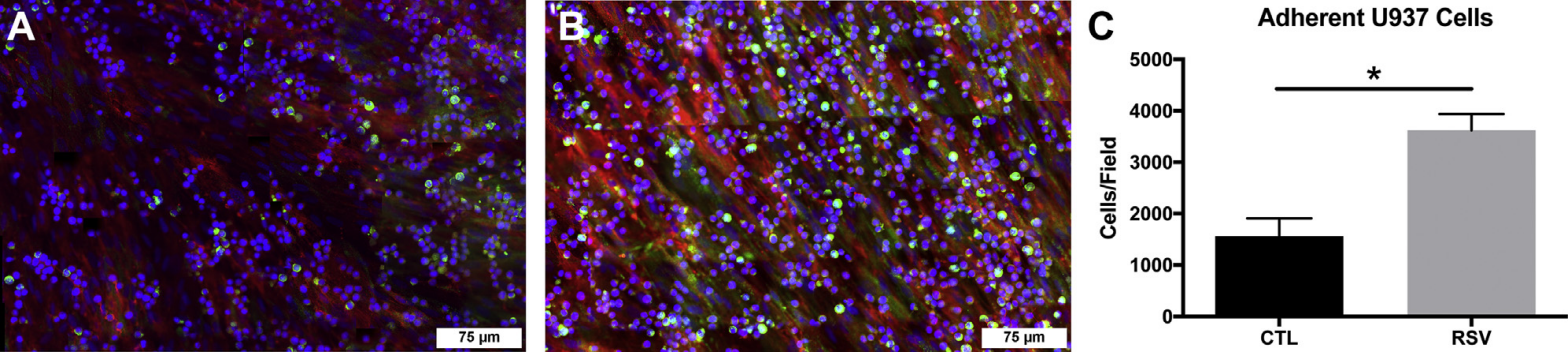
**Human lung fibroblasts cocultured with respiratory syncytial virus (RSV)-infected bronchial epithelial cells produce an extracellular matrix that retains greater numbers of U937 cells.***A–B*, immunofluorescence staining for U937 cells (colabeled with DAPI and anti-CD68, *green*) bound to the HA (*red*)-enriched extracellular matrix demonstrates that the RSV-infected cocultures display greater monocyte retention, which is confirmed by the quantitative analysis (*C*). Panels *A–B* represent cropped regions of interest from a larger 7 × 7 tile scan image with 10% overlap (∗*p* < 0.05, n = 4 per group).

To investigate differences in leukocyte binding properties related to the HA-enriched ECM generated by HLFs cocultured with RSV-infected BECs, U937 cell binding assays were performed using immunofluorescent staining techniques (Fig. 3, *A–B*). HA-enriched ECM generated by HLFs cocultured with RSV-infected BECs displayed 2.3× greater retention of U937 cells compared with uninfected BEC-HLF cocultures in this assay (Fig. 3*C*; *p* < 0.05).

### Coculture of HLFs with BECs following RSV infection did not lead to changes in versican expression by HLFs

**Figure 4:**
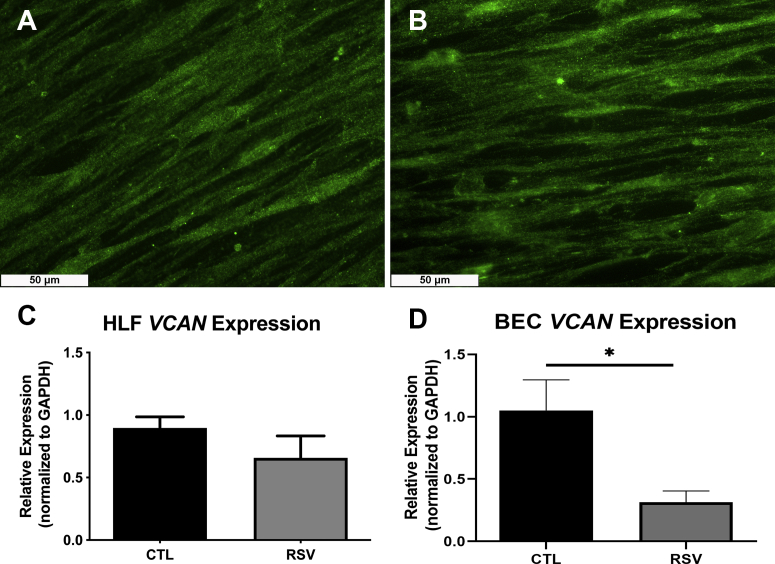
**Versican (*VCAN*) expression by human lung fibroblasts (HLFs) cocultured with bronchial epithelial cells (BECs) with respiratory syncytial virus (RSV) infection.***A–B*, immunofluorescence staining for versican (*green*) is depicted for HLFs cocultured with control BECs and RSV-infected BECs. No differences in *VCAN* expression were observed in HLFs (*C*), whereas the BECs (*D*) demonstrated decreased *VCAN* expression following RSV infection (∗*p* < 0.05; n = 8 per group).

To study the effects of RSV infection on versican expression in our BEC/HLF coculture system, HLFs were cocultured with BECs following RSV or mock infection for 96 h as described above. Comparison of immunofluorescent staining for versican in HLFs cocultured with BECs ± RSV infection did not demonstrate differences in staining patterns or intensity (Fig. 4, *A–B*). Similarly, no differences were detected in HLF expression of versican mRNA (Fig. 4*C*). In contrast, expression of versican by BECs was significantly downregulated in RSV-infected BECs by 48 h (Fig. 4*D*; 67% reduction, *p* < 0.05). The latter finding was unexpected and counter to our original hypothesis that both HA and versican would be upregulated in the setting of RSV infection.

### Evaluation of HA and versican staining in epithelial-specific versican-deficient mice (SPC-Cre(+) Vcan^−/−^)

**Figure 5:**
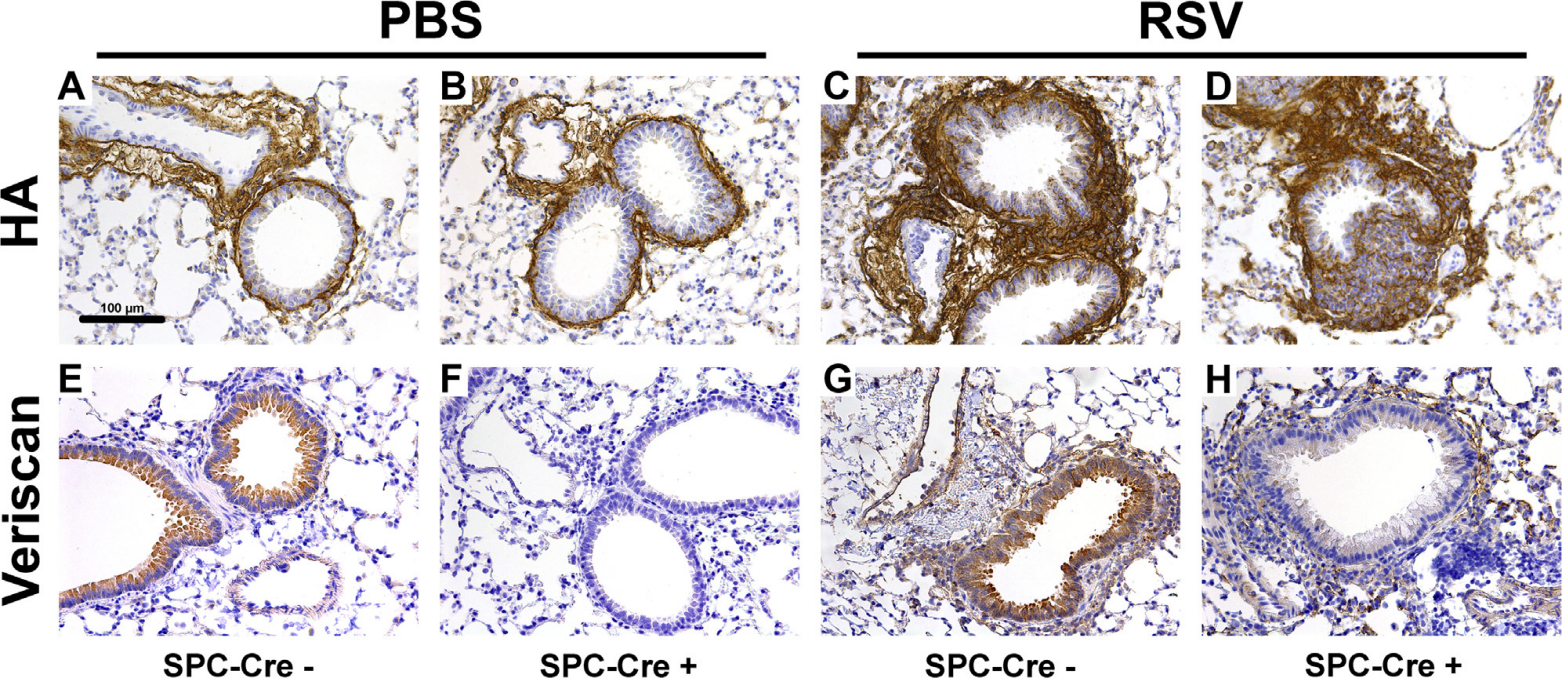
**Hyaluronan (HA) and versican staining in respiratory syncytial virus (RSV)–infected lungs.***A–D*, SPC-Cre(−) and SPC-Cre(+) mice display similar HA in the PBS (*A–B*) and RSV-infected (*C–D*) mice; however, the RSV-infected SPC-Cre(+) mice demonstrate greater leukocyte sequestration (*D*). *E–H*, SPC-Cre(−) display versican only in the epithelium (*E*) compared with the SPC-Cre(+) controls, which is absent (*F*). Versican staining increasing for both after RSV infection (*G–H*) with the SPC-Cre(+) mice again demonstrating greater leukocyte sequestration (*H*).

To further evaluate the effect of decreased versican expression by airway epithelial cells on inflammatory recruitment during RSV infections, mice with the versican gene floxed (*Vcan*^−/−^) were bred with surfactant protein-C Cre (SPC-Cre) mice to establish a colony of mice with lung epithelial-specific knockout of the versican gene. Following infection with RSV or mock infection with PBS, mouse lung histology was examined to observe the presence of HA, versican, and leukocyte sequestration. No difference was observed in HA content between the PBS-treated SPC-Cre(−) versican floxed (Fig. 5*A*) or SPC-Cre(+) versican floxed mice (Fig. 5*B*). Both SPC-Cre(−) and SPC-Cre(+) RSV-infected groups displayed increased staining for HA around the airways and in the endothelium (Fig. 5, *C*–*D*, respectively) without significant differences in staining intensity. SPC-Cre(−) mice demonstrated staining for versican within the airway epithelium (Fig. 5*E*), whereas SPC-Cre(+) mice displayed a distinct absence of staining in this region (Fig. 5*F*). Following RSV infection, SPC-Cre(−) mice displayed more intense versican staining in the airway epithelium than the SPC-Cre(−) PBS-treated controls (Fig. 5*G*), whereas the SPC-Cre(+) RSV-infected mice demonstrated less versican staining compared with SPC-Cre(−) RSV-infected mice. Noteworthy, more versican staining was demonstrated in the airway epithelium of SPC-Cre(+) mice following RSV infection compared with PBS treatment; however, the increased staining may be due to the presence of versican generated by other cell types, including infiltrating leukocytes that are recruited during RSV infection (Fig. 5*H*).

### SPC-Cre(+) Vcan^−/−^ mice display greater leukocyte recruitment in the infected airways and lungs

**Figure 6:**
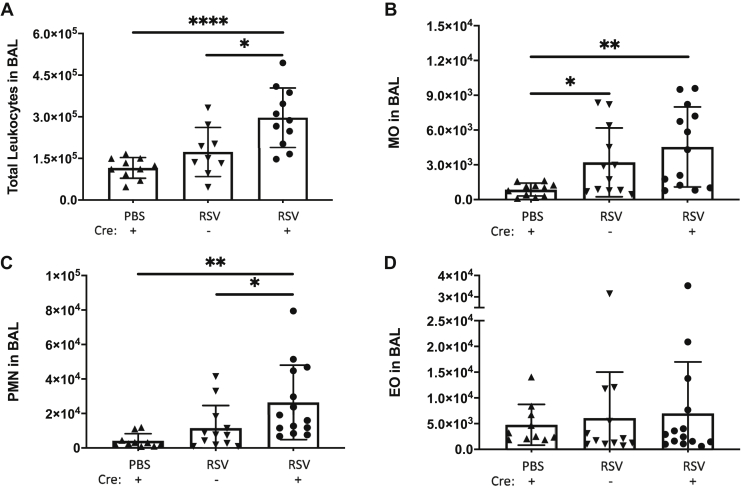
**SPC-Cre versican floxed mice demonstrate increased leukocyte recruitment in the bronchoalveolar lavage fluid.** Bronchiolar lavage was performed on PBS- and respiratory syncytial virus (RSV)-infected mice with the SPC-Cre(+) versican floxed mice showing greater overall recruitment of total leukocytes (*A*) compared with the PBS controls and Cre(−) RSV-infected mice. Monocyte counts (*B*) were similarly increased in both RSV-infected groups. Neutrophil counts (*C*) were increased in both RSV-infected groups but were significantly greater in Cre(+) RSV-infected mice. No differences in eosinophil counts (*D*) were noted across the groups. (∗*p* < 0.05, ∗∗*p* < 0.01, ∗∗∗∗*p* < 0.0001; n = 9 to 11 mice per group)

The increased leukocyte recruitment observed on histology led us to quantify the leukocytes present in the airway and lung tissue. We further broke out the myeloid populations to determine if there was differential recruitment in the absence of epithelial expression of versican. RSV-infected SPC-Cre(+) mice demonstrated a significantly greater leukocyte recruitment in the BALF at 72 h post infection when compared with the PBS controls and infected SPC-Cre(−) littermates (Fig. 6*A*). Differential counts of BALF leukocytes revealed that the monocytes (MOs) were slightly elevated in the SPC-Cre(+) mice (Fig. 6*B*), neutrophils (PMNs) were significantly increased compared with PBS and littermate controls (Fig. 6*C*), and eosinophils (EOs) showed no difference between any of the three groups (Fig. 6*D*). In addition to increased accumulation of leukocytes in the BALF, SPC-Cre(+) RSV-infected mice displayed significantly greater amounts of protein in the BALF, suggesting that the integrity of the airway epithelium may also be compromised in the SPC-Cre(+) mice following RSV infection despite no significant differences in BALF HA concentration (Fig. S1).

**Figure 7:**
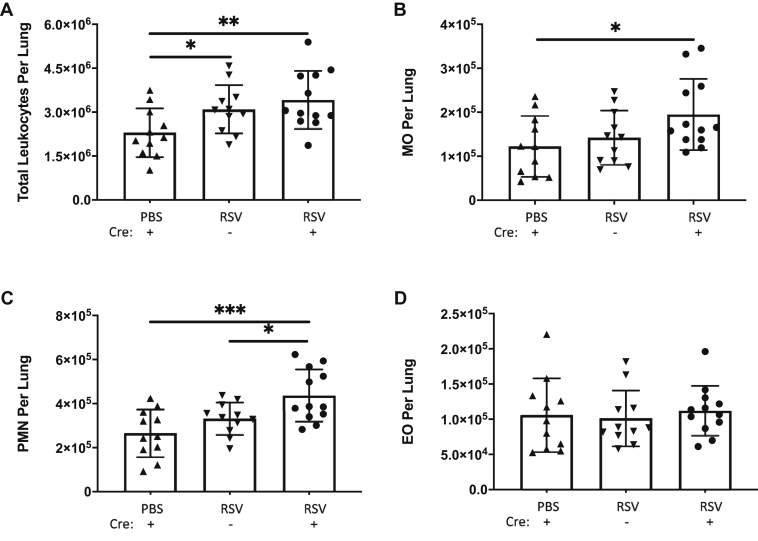
**SPC-Cre versican floxed mice demonstrate increased myeloid recruitment in the lung.** Collagenase digestion was performed on right lung of each PBS- and respiratory syncytial virus (RSV)-inoculated mouse with the SPC-Cre(+) versican floxed mice showing similar increased recruitment of total leukocytes (*A*) compared with the SPC-Cre(−) RSV-infected mice. The SPC-Cre(+) mice displayed increased monocyte (*B*) and neutrophil (*C*) recruitment compared with PBS controls and Cre(−) RSV-infected mice. No differences in eosinophil counts were found across the groups (*D*). (∗*p* < 0.05, ∗∗*p* < 0.01, ∗∗∗*p* < 0.001; n = 11 to 14 mice per group).

RSV infection also elicited increased leukocyte recruitment in the lung tissue of both SPC-Cre(−) and SPC-Cre(+) mice, which was significantly greater than in the PBS-treated controls (Fig. 7*A*). Differential counts of leukocytes in the lung revealed that the MOs were significantly elevated in both SPC-Cre(−) and SPC-Cre(+) mice compared with the PBS controls, but MO counts were not significantly different between SPC-Cre(−) and SPC-Cre(+) mice following RSV infection (Fig. 7*B*). PMN counts were significantly greater in RSV-infected SPC-Cre(+) mice than the PBS and SPC-Cre(−) littermate controls (Fig. 7*C*), whereas the trend in EO recruitment remained similar to that seen in the BALF among the three groups (Fig. 7*D*).

### The absence of epithelial versican leads to differential expression of chemokines, ECM modifiers, and associated cytokines during RSV infection

**Figure 8:**
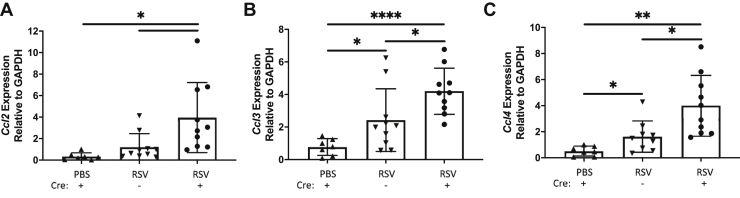
**SPC-Cre versican floxed mice generate increased molecular chemokine markers.** Respiratory syncytial virus–infected SPC-Cre(+) versican floxed mice produce increased molecular expression of the chemokines *Ccl2* (*A*), *Ccl3* (*B*), and *Ccl4* (*C*) compared with the PBS controls and the SPC-Cre(−) respiratory syncytial virus–infected mice, which could account for the increased myeloid recruitment seen in the bronchoalveolar lavage fluid and lung tissue (∗*p* < 0.05, ∗∗*p* < 0.01, ∗∗∗∗*p* < 0.0001; n = 7 to 11 mice per group).

Given that the epithelium influences leukocyte recruitment during viral infection through chemokine signaling (14, 31), we evaluated the expression of *Ccl2*, *Ccl3*, and *Ccl4* in lung homogenates from PBS-treated and RSV-infected mice. The expression of *Ccl2*, also known as monocyte chemoattractant protein 1, was found to be elevated in both RSV-infected SCP-Cre(−) and SPC-Cre(+) mice compared with PBS-treated controls (Fig. 8*A*). Expression of *Ccl2* was significantly greater in SPC-Cre(+) mice compared with SPC-Cre(−) mice following RSV infection. RSV-infected SPC-Cre(+) mice also expressed significantly more *Ccl3* than the PBS-treated controls or the RSV-infected SPC-Cre(−) littermates (Fig. 8*B*). Similar to the expression of *Ccl3*, RSV-infected SPC-Cre(+) mice displayed significantly greater *Ccl4* expression than the PBS-treated mice or RSV-infected SPC-Cre(−) mice (Fig. 8*C*). Noteworthy, the RSV-infected SPC-Cre(−) mice demonstrated significantly greater expression of *Ccl3* and *Ccl4* than the PBS-treated controls consistent with an expected normal immune response. The absence of versican expression in the epithelial cells augmented this response suggesting a counterbalancing role for epithelial-derived versican in the inflammatory response to RSV infection.

**Figure 9:**
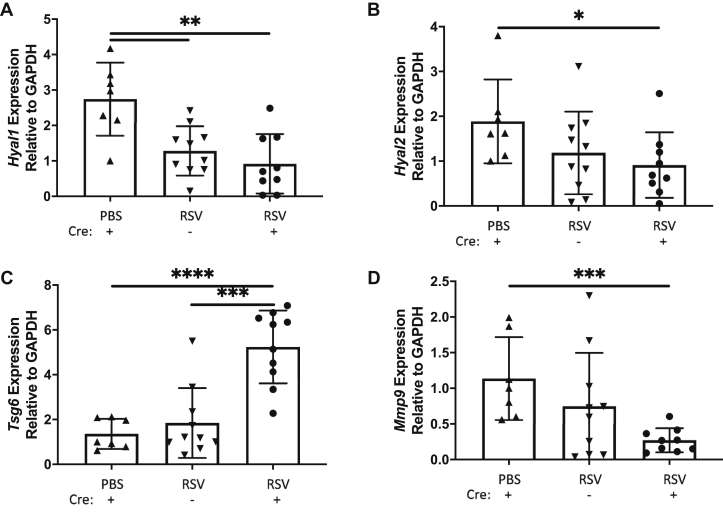
**SPC-Cre versican floxed mice have molecular evidence of altered extracellular matrix kinetics during infection.** Respiratory syncytial virus–infected mice show increased molecular expression of *Hyal1* (*A*) and *Hyal2* (*B*) lending toward decreased hyaluronan metabolism in the infected lung. SPC-Cre(+) versican floxed mice have increased expression of *Tsg6* (*C*) and decreased expression of *Mmp9* (*D*) both of which would impact the extracellular matrix (∗*p* < 0.05, ∗∗*p* < 0.01, ∗∗∗*p* < 0.001, ∗∗∗∗ *p* < 0.0001; n = 7 to 11 mice per group).

In our *ex vivo* human BEC/HLF coculture model system, increased HA accumulation occurred in concert with downregulation of *HYAL1* and *HYAL2*, suggesting that decreased HA turnover may contribute to the increased accumulation of HA. For comparison, we also assessed *Hyal1* and *Hyal2* expression in whole lung homogenates from mice following RSV infection. RSV infection led to a decreased expression of *Hyal1* and *Hyal2* in both SPC-Cre(−) and SPC-Cre(+) mice (Fig. 9, *A–B*, respectively). Both RSV-infected groups of mice demonstrated decreased hyaluronidase expression and increased HA staining compared with the PBS-treated mice. Thus, versican expression may not impact HA metabolism through alteration of *Hyal* expression. We also evaluated, in addition to hyaluronidase expression, the expression of TNFα-stimulated gene-6 (TSG-6), which is a hyaladherin that interacts directly with HA and modifies HA-enriched ECMs impacting their inflammatory properties (32). RSV-infected SPC-Cre(+) mice demonstrated significant increased expression of TSG-6 compared with both SPC-Cre(−) mice and PBS-treated control mice (Fig. 9*C*). Interestingly, there were not significant differences in TSG-6 expression between RSV-infected SPC-Cre(−) mice and PBS-treated mice. We also evaluated matrix metalloproteinase-9 (*Mmp9*) expression in SPC-Cre(+) mice since recent studies have linked versican and MMP9 expression (33). SPC-Cre(+) mice displayed a significant decrease in *Mmp9* expression compared with PBS-treated mice (Fig. 9*D*).

## Discussion

In this study, we have demonstrated that RSV infection alters expression of HA and versican in human BEC/HLF cocultures and that expression of versican in the epithelium is also important for immune regulation in lung epithelial-specific versican-deficient mice. In both model systems, HA accumulation was found to be increased and correlated with downregulation of hyaluronidase expression, suggesting that decreased HA turnover is an important driver of enhanced HA accumulation during RSV infection. Furthermore, in our *ex vivo* human BEC/HLF cocultures, increased HA accumulation corresponded to increased retention of leukocytes in cell adhesion assays. Similarly, increased HA accumulation in the lungs of RSV-infected mice also corresponded to increased leukocyte infiltration. Counter to our initial hypothesis that versican expression would be increased in our *ex vivo* human BEC/HLF coculture system, we found no differences in versican expression by HLFs cocultured with RSV-infected BECs when compared with coculture with noninfected BECs. In contrast, we demonstrated that RSV infection decreases versican expression by differentiated BECs. Further exploration of the role of epithelial-derived versican in our lung epithelium-specific versican-deficient mouse model demonstrated that the versican-deficient mice displayed greater recruitment of inflammatory cells and greater expression of proinflammatory chemokines during RSV infection compared with Cre(−) mice, suggesting an important regulatory role for lung epithelial versican expression during RSV infection.

The role of a HA-enriched ECM in lung inflammation has been the subject of several recent reviews (please refer to 14, 15, 16, 17, 34). The finding of the present study that RSV infection of the BECs leads to the downstream accumulation of HA in the ECM thereby promoting the retention of leukocytes is novel; however, this finding is consistent with other studies that have evaluated the role of HA in lung infections. For example, previous studies have shown that HLFs treated with poly I:C also form a HA-enriched ECM by shifting HA accumulation from the culture media to the cell layer and forming an ECM that more avidly binds leukocytes (21). Interestingly, the same study also demonstrated a critical role for the accumulation of versican in promoting leukocyte binding in poly I:C-treated HLFs. More recently, Bell and colleagues reported that HA-enriched ECMs play a critical role in inflammatory recruitment in a murine model of influenza infection (35). In that study, the authors demonstrated that influenza infection leads to persistent induction of *Has2* across multiple cells types resulting in increased accumulation of HA and enhanced recruitment of leukocytes with deleterious effects on pulmonary function that last far beyond the clearance of the influenza virus. Strikingly, treatment with exogenous hyaluronidase before the peak of illness greatly decreased the weight loss and improved the pulmonary function of the influenza-infected mice. The authors further implicated a potential role for heavy chain–HA suggesting the importance of TSG-6 activity in the modulation of the inflammatory process induced by the altered HA accumulation (35). Similarly, we also observed increased TSG-6 expression in our epithelial-specific versican-deficient mice following RSV infection, yet we have not further characterized its role in this study. The increased expression of TSG-6 in the versican-deficient mice is very interesting given that both molecules are known to directly bind to HA and influence inflammatory recruitment (32). Further evaluation of TSG-6 expression and/or activity in this model system may provide additional insight into the formation of an altered HA-rich ECM during RSV infection and will be an important area of future study.

To date, there are few examples in the literature that describe the effect of RSV infection on the formation of HA-enriched ECMs in the context of lung inflammation. Recently, members of our group reported that direct RSV infection of cultured HLFs results in the increased accumulation of HA within the cell layer promoting the retention of mast cells (25). In contrast to the results reported in the present study, RSV-infected HLFs significantly upregulated *HAS2* and *HAS3* expression while also downregulating *HYAL2* expression suggesting that increased HA accumulation was the result of both enhanced production and decreased turnover in HLFs. In comparison, the present study demonstrates reductions in both *HYAL1* and *HYAL2* without significant differences in *HAS2* or *HAS3* expression by HLFs cocultured with RSV-infected BECs. Although the altered balance of HA syntheses and degradation may be sufficient to explain the greater accumulation of HA during RSV infection, it is important to note that HASes are additionally subject to post-translational modifications that can also affect their activity and alter HA production (36). Furthermore, it is worth noting that other factors may also influence hyaluronidase activity, including expression of other recently identified HA degradation enzymes such as HYBID and TMEM2, which were not evaluated in the present study (37). Another interesting comparison between these studies is that the distribution of HA size contained within the cell layer was skewed toward a HMW-HA profile in HLFs directly infected with RSV compared with the HA contained within the cell layer of HLFs within our BEC/HLF coculture model system, which demonstrated LMW-HA profiles. Taken together, these data suggest that BECs regardless of RSV infection may influence HA processing and turnover by HLFs thereby modifying the distribution of HA contained within the ECM. The latter is potentially of significant interest given that HMW-HA and LMW-HA have been shown to demonstrate different effects on inflammatory recruitment and activation (38).

In addition to the alterations observed in HA accumulation in our different models of lung infection, alterations in HA-binding partners such as versican have also been shown to modulate the ability of the ECM to regulate inflammatory changes during lung infection (26). Versican is not normally expressed in the healthy mature lung; however, it is rapidly induced during lung injury or infection (39, 40). Our initial hypothesis that versican would be upregulated in the setting of RSV infection was largely based on the findings reported by Kang *et al.* (30), which demonstrated that mice treated with intratracheal instillation of poly I:C displayed increases in both HA and versican accumulation in the peribronchial and perivascular regions that correlated with the onset of inflammatory changes observed in the lung. Interestingly, when similar poly I:C treatments were performed in mice globally deficient in versican as a result of an inducible *Vcan*^−/−^ system, the increased accumulation of versican, HA, and inflammatory changes were all greatly attenuated, suggesting an important role for versican as an inflammatory mediator during acute lung injury. This concept was further underscored in experiments demonstrating that HLFs obtained from versican-deficient mice did not exhibit increased HA-dependent binding of leukocytes following treatment with poly I:C in *ex vivo* binding studies (30). In contrast to the findings reported by Kang *et al.*, in our study, epithelial-specific versican-deficient mice displayed enhanced recruitment of inflammatory cells in both the lung and airway lumen suggesting that epithelial-derived versican may be an important counterbalance that helps regulate airway inflammation in the setting of infection. Separate studies have shown that MMP9 is responsible for cleaving transforming growth factor B from the ECM modulating inflammation and cellular repair, which may be a factor in the enhanced inflammatory response observed in the SPC-Cre(+) mice (41).

Beyond the inherent differences between the epithelial-derived versican knockout mice and the inducible global versican knockout mice, it is important to note that there are also significant differences between poly I:C treatment and RSV infection. Poly I:C is a TLR3 antagonist, which is not highly expressed on the cellular surface at homeostasis, residing mainly in internal vesicles (42). This may lead to a delay in immune signaling and subsequent reduced expression of versican in the leukocytes, which may impact MO phenotype and activity (43). In addition to TLR3 signaling, the fusion protein of RSV also signals through TLR4, eliciting further immunological signaling and PMN activity, contributing to the increased myeloid recruitment seen in the RSV-infected mice (44). In a separate study, using TLR4 knockout mice, Chang *et al*. (39) demonstrated that TLR4 signaling is a major determinant of MO activity, where the absence of the pathogen receptor led to a decrease in HA and versican expression. Another important consideration is that the epithelium significantly contributes to immunological activity during infection through a variety of signaling mechanisms (45). The lack of versican may facilitate or potentiate epithelial damage, which in turn could lead to increased release of cytokines, chemokines, and alarmins driving greater leukocyte recruitment observed in the SPC-Cre(+) mice. Thus, the persistence of the RSV infection may perpetuate the cycle of epithelial damage and increased leukocyte recruitment in a more robust way than a single dose of poly I:C in this context.

A significant strength of the present study is the use of an *ex vivo* differentiated primary human BEC/HLF coculture model system, which allowed for the cross talk of BECs and HLFs to be modeled in the setting of RSV infection. This model system better approximates the *in vivo* situation than either cell type in isolation given that the primary cellular target of RSV is the ciliated BECs and the primary effector cells of ECM production are the fibroblasts. Through this model, we were able to observe the downstream effects of RSV infection on the regulation of HLF deposition of HA and versican in the ECM. A significant limitation of this study is that we do not know what signal(s) produced by the BECs are driving the enhanced accumulation of HA by the HLFs because examination of the coculture media would not allow for isolating soluble mediators to one cell type or the other. This in itself is a very interesting area of investigation that will require further characterization in future studies and may provide targets for therapeutic strategies. Similarly, we did not evaluate the role of reactive oxygen species in our model system which may also be an important therapeutic target given that reactive oxygen species are known to facilitate the fragmentation of HA during inflammation (46). The use of a tissue-specific knockout mouse is another strength of the present study. Given that it is embryonic lethal, use of a global versican knockout is not feasible (47). Other inducible versican-deficient mouse models have been developed in order to circumvent this limitation and have provided valuable information on the role of versican during lung inflammation (30). However, available inducible versican-deficient mice also have the inherent limitations of the need for pretreatment with tamoxifen and lack tissue specificity, whereas the mice used in this study display tissue-specific knockout of versican in the airway epithelium. An important caveat of this model is that the knockout is constitutive once the airway epithelial cells are differentiated and begin to express the SPC gene at E10.5 directing recombination in all airway and alveolar epithelial cells (48). Therefore, the fact that any homeostatic role that versican may play in the airway epithelium or the alveolar epithelium at baseline is also likely to be affected in these mice and must be taken into account when interpreting the results of this study.

In conclusion, we have demonstrated that alterations of HA accumulation and versican expression play an important role in the inflammatory process caused by RSV infection. Accumulation of HA following RSV infection is enhanced and may contribute to the establishment of an ECM that promotes greater recruitment and retention of leukocytes, thereby contributing to the overall inflammatory process. In contrast, genetic deletion of epithelial-derived versican expression also promotes airway inflammation in RSV-infected mice, suggesting that versican likely plays a more complex role in the regulation of airway inflammation in this context. These findings are of significant interest given that, traditionally, versican expression has been thought to track with HA accumulation during lung inflammation. Our findings suggest that versican may play a counterbalancing role to the proinflammatory effects of an HA-enriched ECM during lung injury. Whether the absence of epithelial-derived versican allows HA to interact with other proinflammatory molecules to promote inflammation or conversely versican independently exhibits anti-inflammatory properties to contain the inflammatory response remains an open question and will require additional future studies. Greater knowledge of the pro- or anti-inflammatory properties of versican will lead to better understanding of how versican and other ECM constituents such as HA contribute to the inflammatory process during RSV infection and may be more broadly applicable to other respiratory viruses including SARS-CoV-2.

## Experimental procedures

### Establishment of *ex vivo* human BEC–HLF cocultures

#### Human subjects

Following informed consent, pediatric subjects (ages 6–18 years) undergoing elective surgery requiring endotracheal intubation and general anesthesia at Seattle Children’s Hospital were recruited as part of an ongoing Institutional Review Board–approved study that abides by the Declaration of Helsinki principles. Subjects were screened for a clinical history of asthma, recurrent wheezing, chronic cough, and/or preterm delivery. Written informed consent was obtained from a parent or legal guardian for all subjects below the age of 18 years, or from the subject if age 18 years. Written assent was obtained for children ≥ age 10 years. Only BECs obtained from nonasthmatic subjects were included in the present study.

#### Isolation and differentiation of primary human BECs

After endotracheal intubation, primary BECs were obtained *via* three blind bronchial brushings using a 4-mm unsheathed Harrell bronchial brush as previously described (49). BECs were seeded into separate T-25 flasks and proliferated under submerged conditions in PneumaCult-Ex media (StemCell) containing gentamicin and amphotericin B and further supplemented with penicillin-streptomycin (100 μg/ml; Invitrogen). Fluconazole (25 μg/ml) was added to primary cultures for the first 96 h, after which medium was aspirated and replaced with standard PneumaCult-Ex. Culture media was changed every 48 h until the cells reached ∼70% to 90% confluence, at which point they were either passaged or cryopreserved.

BECs (passage ≤3) were seeded into type I collagen–coated transwell plates (12 mm, 0.4-μm pore Costar, Corning Life Sciences) at a concentration of 100,000 cells per transwell and were maintained in submerged culture until confluence was reached. Once confluent, the cell cultures were switched to an air–liquid interface (ALI) by adding PneumaCult ALI Media to the basal transwell chamber only. BECs were differentiated at an ALI for 21 days with media changes occurring every other day through the initiation of experiments.

#### Bronchial epithelial cell–lung fibroblast cocultures

Primary HLFs from a healthy pediatric donor were obtained from a commercial vender (Lonza). HLFs from the same passage (passage 6) were used for all coculture experiments. HLFs were seeded at a density of ∼2500 cells/cm^2^ in 12-well plates coated with type I collagen and incubated for 3 to 4 days to achieve a confluent monolayer prior to initiation of BEC/HLF cocultures. HLF cultures were maintained in Fibroblast Growth Media BulletKit (FGM-2) with media changes occurring at 48-h intervals. At experiment day 0, the HLF media was replaced with coculture media (1:1 FGM-2 and PneumaCult ALI Maintenance Media). ALI transwells containing BECs were placed in coculture with the HLFs at experimental day 0 by transferring the transwell inserts to the well plates containing the established HLF cultures. The differentiated BEC cultures were in close proximity to the submerged HLF cultures and shared the same media. Coculture media changes occurred daily.

### RSV infection of human BEC–HLF cocultures

Differentiated BEC cultures were infected with RSV Line 19 by exposing the apical surface to media containing RSV at multiplicity of infection of 1 for 2 h after which the apical media was removed and discarded. RSV-infected BECs were placed in coculture with HLFs as described above and maintained for a 96-h exposure after which materials were collected for analysis and endpoint assays were performed.

### RNA extraction and real-time PCR for human BEC–HLF cocultures

Messenger RNA was isolated from HLFs cocultured with BECs. Three wells from each experimental condition were harvested and pooled to isolate RNA according to manufacturer recommendations (RNAqueous kit, Ambion, Applied Biosystems). RNA concentration and quality were determined using the NanoDrop One Microvolume UV-Vis Spectrophotometer (Thermo Fisher Scientific). RNA samples were reverse transcribed using the SuperScript VILO cDNA Synthesis Kit (Life Technologies). Quantitative real-time PCR was performed using validated TaqMan probes (Life Technologies) for hyaluronan synthase (*HAS*)*1* (Hs00987418_m1), *HAS2* (Hs00193435_m1), *HAS3* (Hs00193436_m1), *hyaluronidase (HYAL)1* (Hs00201046_m1), *HYAL2* (Hs01117343_g1), *CD44* (Hs01075861_m1), versican (*VCAN*, Hs00171642_m1), and glyceraldehyde 3-phosphate dehydrogenase (*GAPDH*, Hs02758991_g1). Assays were performed using the TaqMan Fast Advanced Master Mix reagents and the Applied Biosystems StepOnePlus Real-Time PCR System (Life Technologies).

### Immunohistochemistry for human BEC–HLF cocultures

Prior to seeding HLFs, sterilized 12-mm round glass coverslips were placed in the bottom of replicate chambers of the 12-well plates. Following 96 h of coculture, the coverslips were carefully removed, fixed, and permeabilized with 50:50 methanol/acetone at 20 °C for 10 min and stained with biotinylated hyaluronan binding protein (HABP) primary (2.5 μg/ml, Millipore) and a streptavidin conjugated Alexa Fluor 568 secondary (1:1000, Thermo Fisher). Separate staining was performed for versican (1:100, ab177480 Abcam) and a goat anti-rabbit Alexa Fluor 488 secondary antibody (1:1000, Thermo Fisher). Imaging was obtained with conventional epi-fluorescence microscopy (DM6000B, Leica).

### Leukocyte binding assays

Subsets of cocultured HLFs were used to assess the ability of the secreted ECM to bind U937 cells (ATCC). Cells were washed twice in phenol-free media and resuspended (3 × 10^6^ cells/ml). HLF wells were washed with RPMI. Following this, 1.0 ml of the leukocyte suspension was added to the wells and allowed to bind at 4 °C for 90 min. Cultures were washed five times in cold RPMI to remove nonadherent cells. U937 cell binding was imaged with fluorescent microscopy following fixation on coverslips. Fixed U937 cell binding assays followed the same IHC protocol as above for HABP staining with the modification of a primary antibody against the monocyte marker CD68 (1:200, mouse KP-1 monoclonal, Abcam) and a secondary antibody (1:1000, donkey anti-mouse Alexa Fluor 488, Thermo Fisher) at the corresponding steps in the staining protocol. Cell counts were performed using ImageJ software (NIH, Bethesda, MD).

### Quantitative analysis of HA content and fragment size

HA content and hydrodynamic size were assessed using a modification of previously reported methods (50, 51). Media and cell layer samples were isolated separately and digested with pronase (300 μg/ml, Roche) in 0.5 M Tris buffer (pH 6.5) overnight at 37 °C. Following digestion, the pronase was heat inactivated by incubation at 100 °C for 20 min. Media and cell layer concentrations of HA were measured using an enzyme-linked immunosorbent assay (ELISA) from R&D Systems (kit DY3614-05). To determine the hydrodynamic size of HA fragments contained within the samples, equal amounts of HA were applied to an S-1000 column (GE Healthcare) in a 0.5 M sodium acetate buffer containing 0.025% Chaps, 0.02% sodium azide at pH 7.0. Samples were collected using a microtube fraction collector (Model 2110, Bio-Rad) and were analyzed separately using the HA ELISA described above.

### Description of mice

SPC-Cre(+) female mice (48) were bred onto a versican floxed C57BL/6 background (30) for six generations. The resulting male and female 8- to 10-week-old SPC-Cre(+) and SPC-Cre(−) versican floxed mice were housed at the Benaroya Research Institute’s (BRI) Biosafety Level-2 mouse facility where they were given an intranasal inoculation of 40 μl of PBS or 40 μl of RSV (containing 1.5 × 10^5^ plaque-forming units of Line-19 RSV) then euthanized with 1.5 ml of avertin at 18 h or 72 h. All animal procedures were approved by the BRI Institutional Animal Care and Use Committee.

### Bronchiolar lavage

Bronchiolar lavage was performed collecting three separate samples of 0.8 ml from each mouse. Lavages were stored on wet ice until centrifuged at 1500 rpm for 5 min to separate the cells. After collection, the cells were treated in ammonium-chloride-potassium lysis buffer for 10 min at 4 °C to remove the red blood cells, then washed in PBS.

### Whole lung cell isolation

The right lung of each mouse was perfused with 3 ml of PBS, excised from the bronchus, then placed in a 12-well plate containing PBS on wet ice. Within 3 h, lungs were minced for digestion, transferred to a 12-well plate containing 2 ml per sample of a collagenase mix (isolated from *Clostridium histolyticum* [Sigma]) and incubated at 37 °C for 45 to 60 min. After incubation, the tissue was regurgitated through a 16-gauge needle five times to further break it down, then pressed through a 40-μm filter, which was washed with PBS. The tissue slurry was centrifuged at 1500 rpm for 5 min, the supernatant decanted, and the collected cells were then treated with ammonium-chloride-potassium for 10 min at 4 °C, and then washed in PBS.

### Flow cytometry analysis

The cells yielded from the bronchiolar lavage and lung were stained using fluorescent-labeled antibodies at 4 °C in the dark for 20 min, washed with PBS, and then examined on the BD Biosciences LSR II platform in the BRI flow cytometry core. Leukocytes were stained for CD11b, CD11c, CD45.2, CD103, Ly6C, Ly6G, MHC-II, SiglecF (all BioLegend), and Viability (eBioscience).

### RNA extraction and real-time PCR for mouse studies

Total RNA was isolated from the upper left lung lobe of mice from each test group. After PBS perfusion, the lung lobe was snap frozen on dry ice and RNA was later isolated utilizing the Nucleospin RNA isolation kit (Takara), then converted to cDNA for quantification. Quantitative real-time PCR was performed using validated TaqMan probes (Life Technologies) for *Gapdh* (Mm99999915_g1), *Ccl2* (SMm00441242_m1), *Ccl3* (Mm00441259_g1), *Ccl4* (Mm00443111_m1), *Has1* (Mm03048195_m1), *Has2* (Mm00515089_m1), *Hyal1* (Mm00476206_m1), *Hyal2* (Mm01230689_g1), *Il6* (Mm00446190_m1), *Mmp9* (Mm00442991_m1), and *Tnfaip6* (Mm00493736_m1). Assays were performed using the TaqMan Fast Advanced Master Mix reagents and the TaqMan primer/probe sets on the Thermo Fisher Scientific QuantStudio-5 real-time qPCR platform.

### Immunohistochemistry for mouse studies

The lower left lung was inflated with a 50/50 mixture of OCT–PBS and placed in 10% formalin for histological preparation by the BRI Histology Core utilizing a biotinylated HABP (prepared in-house), then counterstained with streptavidin HBR label (Biocare Medical). Versican immunohistochemistry required pretreatment using heat-mediated antigen retrieval with low pH for 10 min and digestion with 0.2 U/ml chondroitinase ABC (Sigma, #C3667) in a buffer containing 18 mM Tris, 1 mM sodium acetate, and 1 mg/ml BSA pH 8.0 for 1 h at 37 °C. After digestion, lung sections were incubated for 1 h with 2 μg/ml rabbit anti-mouse versican-GAG β domain (EMD Millipore, #AB1033) in bond antibody diluent, and detection was performed using the Bond Polymer Refine Detection kit (Leica Biosystems).

### Statistical analysis

Analyses of PCR results were performed using GenEx version 6.0.5 (MultiD Analyses AB) based on previously described methods (52). For all other data, the unpaired *t* test was used for comparisons that were normally distributed within each group. For nonnormally distributed data, the Mann-Whitney test was used. Statistical analyses were performed using Prism 8.0 software (Graph-Pad Software Inc). Statistical significance was set at *p* < 0.05.

## Data availability

The raw data supporting the conclusions of this article will be made available by the authors, without undue reservation, to any qualified researcher.

## Conflicts of interest

The authors declare that they have no conflicts of interest with the contents of this article.
