# Supplementary material for: Loss of versican and production of hyaluronan in lung epithelial cells are associated with airway inflammation during RSV infection
Source: J Biol Chem. 2020 Nov 21;296:100076. doi: 10.1074/jbc.RA120.016196 (PMC7949086; doi:10.1074/jbc.RA120.016196)
Supplement: Figures S1 [file mmc1.pdf]

Supplemental Data

Loss of versican and production of hyaluronan in lung epithelial cells are associated with airway inflammation during RSV infection

Gerald G. Kellar, MS, MLS(ASCP)SBB,[1,2,3] Kaitlyn A. Barrow, BA,[4] Lucille M. Rich, BS,[4] Jason S. Debley, MD, MPH,[4,5] Thomas N. Wight, PhD,[2] Steven F. Ziegler, PhD,[2,3] Stephen R. Reeves, MD, PhD[4,5]

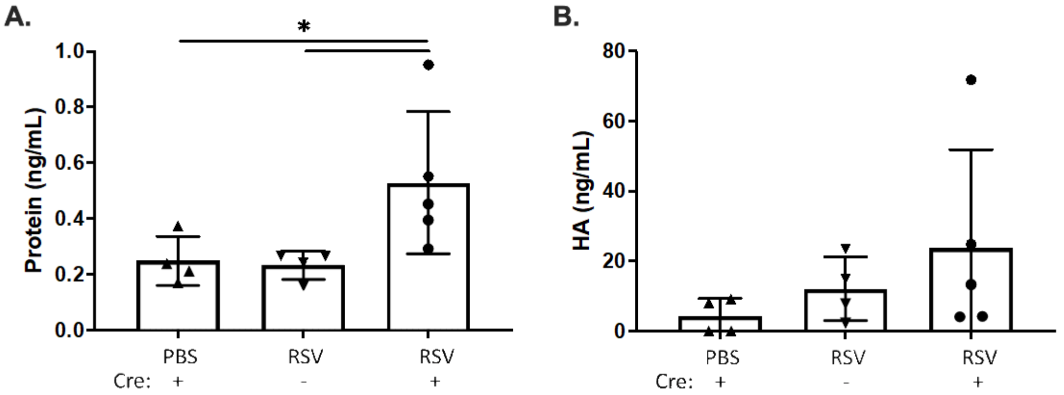

Figure S1. **SPC-Cre versican floxed mice have increased protein but not HA content in the BALF.** ***A***, The SPC-Cre(+) mice demonstrate greater protein content in their BALF despite not showing a greater quantity of HA (***B***). (*$p < 0.05$)
